# Biological Impact of Target Fragments on Proton Treatment Plans: An Analysis Based on the Current Cross-Section Data and a Full Mixed Field Approach

**DOI:** 10.3390/cancers13194768

**Published:** 2021-09-24

**Authors:** Elettra Valentina Bellinzona, Leszek Grzanka, Andrea Attili, Francesco Tommasino, Thomas Friedrich, Michael Krämer, Michael Scholz, Giuseppe Battistoni, Alessia Embriaco, Davide Chiappara, Giuseppe A. P. Cirrone, Giada Petringa, Marco Durante, Emanuele Scifoni

**Affiliations:** 1 Trento Institute for Fundamental Physics and Applications (TIFPA), National Institute for Nuclear Physics, (INFN), 38123 Trento, Italy; elettra.bellinzona@tifpa.infn.it (E.V.B.); francesco.tommasino@unitn.it (F.T.); 2 Department of Physics, University of Trento, 38123 Trento, Italy; giuseppe.battistoni@tifpa.infn.it; 3 The Department of Radiation Research and Proton Radiotherapy, Institute of Nuclear Physics, Polish Academy of Sciences, 31-342 Krakow, Poland; leszek.grzanka@ifj.edu.pl; 4 “Roma Tre” Section, INFN—National Institute for Nuclear Physics, 00146 Roma, Italy; andrea.attili@roma3.infn.it; 5 Department of Biophysics, GSI Helmholtzzentrum für Schwerionenforschung, 64291 Darmstadt, Germany; T.Friedrich@gsi.de (T.F.); m.kraemer@gsi.de (M.K.); m.scholz@gsi.de (M.S.); m.durante@gsi.de (M.D.); 6 “Pavia” Section, INFN—National Institute for Nuclear Physics, 6-27100 Pavia, Italy; Alessia.Embriaco@pv.infn.it; 7 Laboratori Nazionali del Sud, INFN—National Institute for Nuclear Physics, 95125 Catania, Italy; davide.chiappara@gmail.com (D.C.); pablo.cirrone@lns.infn.it (G.A.P.C.); giada.petringa@lns.infn.it (G.P.); 8 Institut für Physik Kondensierter Materie, Technische Universität, 64289 Darmstadt, Germany

**Keywords:** proton therapy, relative biological effectiveness (RBE), mixed field model, Monte Carlo, TOPAS, biophysical dose-response models

## Abstract

**Simple Summary:**

Proton therapy is now an established external radiotherapy modality for cancer treatment. Clinical routine currently neglects the radiobiological impact of nuclear target fragments even if experimental evidences show a significant enhancement in cell-killing effect due to secondary particles. This paper quantifies the contribution of proton target fragments of different charge in different irradiation scenarios and compares the computationally predicted corrections to the overall biological dose with experimental data.

**Abstract:**

Clinical routine in proton therapy currently neglects the radiobiological impact of nuclear target fragments generated by proton beams. This is partially due to the difficult characterization of the irradiation field. The detection of low energetic fragments, secondary protons and fragments, is in fact challenging due to their very short range. However, considering their low residual energy and therefore high LET, the possible contribution of such heavy particles to the overall biological effect could be not negligible. In this context, we performed a systematic analysis aimed at an explicit assessment of the RBE (relative biological effectiveness, i.e., the ratio of photon to proton physical dose needed to achieve the same biological effect) contribution of target fragments in the biological dose calculations of proton fields. The TOPAS Monte Carlo code has been used to characterize the radiation field, i.e., for the scoring of primary protons and fragments in an exemplary water target. TRiP98, in combination with LEM IV RBE tables, was then employed to evaluate the RBE with a mixed field approach accounting for fragments’ contributions. The results were compared with that obtained by considering only primary protons for the pristine beam and spread out Bragg peak (SOBP) irradiations, in order to estimate the relative weight of target fragments to the overall RBE. A sensitivity analysis of the secondary particles production cross-sections to the biological dose has been also carried out in this study. Finally, our modeling approach was applied to the analysis of a selection of cell survival and RBE data extracted from published in vitro studies. Our results indicate that, for high energy proton beams, the main contribution to the biological effect due to the secondary particles can be attributed to secondary protons, while the contribution of heavier fragments is mainly due to helium. The impact of target fragments on the biological dose is maximized in the entrance channels and for small α/β values. When applied to the description of survival data, model predictions including all fragments allowed better agreement to experimental data at high energies, while a minor effect was observed in the peak region. An improved description was also obtained when including the fragments’ contribution to describe RBE data. Overall, this analysis indicates that a minor contribution can be expected to the overall RBE resulting from target fragments. However, considering the fragmentation effects can improve the agreement with experimental data for high energy proton beams.

## 1. Introduction

Proton therapy (PT) is establishing as a clinical treatment procedure in radiotherapy, with indications for specific types of cancer which are difficult to treat with surgery or conventional radiotherapy using photons [1,2,3,4]. Compared to X-rays, PT shows a particular advantage, especially in terms of physical dose distribution (i.e., the Bragg peak curve) [5].

At the same time, the biological effectiveness of protons is assumed to be similar to that of X-rays in the beam entrance region, considering a constant RBE value of 1.1, while several experimental evidences show that it increases substantially in the peak region as protons slow down and LET (linear energy transfer, i.e., the electronic stopping power) increases. This can be quantified by the RBE parameter. Despite the extensive research efforts that have been dedicated over the years to evaluate the potential benefit of including also in therapeutic treatments a variable RBE, a constant RBE value of 1.1 is currently adopted in the clinics [6,7,8]. Nevertheless, in vitro studies show a remarkable difference in RBE, especially when looking at the survival level measured at the end of protons’ range, which significantly exceeds the 1.1 value, with a pronounced dependence on tissue type [9,10,11]. Most of the research in the field is therefore concentrated on the description of the distal end and the fall-off region of the Bragg peak [12,13,14].

However, a fragmentation induced build-up effect has been shown in the entrance channel due to secondary protons [15] and several in vitro measurements also point to a larger RBE than expected in the entrance region [3,10,16,17]. The reasons for such deviation from the constant RBE factor could be partially attributed to an insufficient description of the radiation field in the entrance channel. The contribution resulting from the fragmentation of the target nuclei is, indeed, usually neglected.

An accurate description of the total nuclear cross-sections resulting from the interactions of the proton beam with target nuclei may represent a remarkable feature for biologically oriented ion-therapy planning. Nuclear cross-sections determine the fluence decrease of primary particles, translating into a spectrum of secondary particles with different ranges and scattering angles, realizing a peculiar complex radiation field with specific biological effect. This holds, in principle, for both projectile and target fragmentation.

For instance, projectile fragmentation has an obvious impact on the depth-dose profile of a carbon ion beam and therefore has to be taken into account by the planning software. This would clearly apply also to alternative candidate ions for charged particle therapy, as it is the case for helium and oxygen [18,19]. Along with the variable RBE attributed to the primary beam, fragments are also considered with their contributions to physical dose and to RBE [20,21]. A useful illustrative study of the impact of nuclear fragmentation description in biologically weighted dose profiles in the case of carbon ions has been performed in [22].

In proton treatments, the physical fluence attenuation of the primary beam due to nuclear interactions is included in the planning software programs, and the contribution to the physical dose from secondary protons is taken into account with different parameterizations [23,24,25]. On the contrary, heavier target fragments are usually neglected. The recent advent of Monte Carlo (MC) TPS in the clinics contributed to partially improve the description of nuclear interactions [26].

The potential contribution of target fragments to the overall biological effect was already addressed in the past. It is worth mentioning the modeling study by Cucinotta et al. [27] as well as the MC investigation performed more recently by Grassberger and Paganetti [28]. However, an accurate assessment of the biological effects of target fragments was hindered also by the very limited availability of experimental cross-sections in the range of energy of interest.

These target fragments are generated by inelastic interactions of the primary protons with the target nuclei and therefore have low kinetic energies and range. Target fragments are thus expected to have high LET values and thus an increased RBE [17]. The production cross-sections and in particular the kinetic energy dependent fragments’ yields in the therapeutic energy range are still affected by large uncertainties.

Recently, the research activity within the MoVe-IT (Modeling and Verification for Ion beam Treatment planning [29]) collaboration was including in its focus the evaluation of the biological impact of such target fragments; in parallel, the FOOT (Fragmentation Of Target) [30,31,32] experiment started, aiming at the production of a large dataset of fragmentation cross-sections that would fill the current gaps.

Previous modeling work in the field [33] addressed this problem with a phenomenological approach, including the description of fragments until Z = 2 in the mixed radiation field. A recent work from our group was instead investigating the impact of target fragments on biological dose using a simple dose-averaged LET modification through different parametric models [34].

This paper is dedicated to the systematic analysis of target fragments contribution to the RBE of a proton beam, and therefore to its biological dose in a water target. Based on the combination of different research tools, we evaluated the dosimetric and RBE contribution of secondary protons and heavier fragments with respect to the primary protons. The analysis is finally applied to the description of experimental data in terms of in vitro clonogenic cell survival and RBE.

## 2. Materials and Methods

The analysis is based on the consideration of the possible contributions to the biological dose starting from a primary proton beam irradiation, and analyzing three simulation scenarios: primary protons only, primary and secondary protons and the full particle spectrum (primary and secondary protons and all target fragments). Primary protons are defined as protons which haven’t gone through a nuclear reaction, where fluence attenuation is properly accounted to match the clinical calculus. Both secondary protons components have been accounted: the one produced as a recoil of elastic interaction of primary proton with hydrogen and the one produced as secondary fragments created by nuclear interactions of primary proton, mainly with oxygen.

The particle fluence spectra were obtained, for each scenario, with the TOPAS MC simulations [35] (TOPAS version 3.3), then converted into a binary format suitable for the TRiP98 TPS [20,36,37] (SPC (http://bio.gsi.de/DOCS/TRiP98/DOCS/trip98fmtspc.html, accessed on 31 July 2021)) by using the tool described in Section 2.1 and therefore employed to calculate the biological dose by using the mixed field approach implemented in TRiP98 TPS. TOPAS is a wrapper of the Geant4 simulation toolkit. Version 3.3 contains embedded libraries and data files from Geant4 version 10.05.p01. TRiP98 is instead a research tool which was developed and employed clinically for the pilot project at GSI and that continues to be expanded and used for research purposes in several centers in Europe. The depth dose distributions are stored for each energy (DDD (http://bio.gsi.de/DOCS/TRiP98/PRO/DOCS/trip98cmdddd.html, accessed on 31 July 2021)). First, the case of a mono-energetic beams is analyzed at various initial kinetic energies and depths for an exemplary case of normal and tumor tissues (α/β = 2 Gy with LEM threshold dose of $D_{t}$ = 8 Gy and α/β = 10 Gy, $D_{t}$ = 14 Gy respectively) [38]. Two SOBPs have been therefore studied and compared with experimental data. To study the radiobiological impact of target fragments, proton beams with different initial kinetic energies have been simulated by TOPAS in liquid water. In order to deal with the low statistics of secondary particles, due to their low production cross-section σ, and their short range and low energy, a particular track-length fluence scoring has been adopted to score the fluence-energy spectra, as described in Section 2.1.

### 2.1. Spectra Simulation

As mentioned above, the TOPAS code has been used to create spectra tables and depth-dose distributions [39].

**Figure 1:**
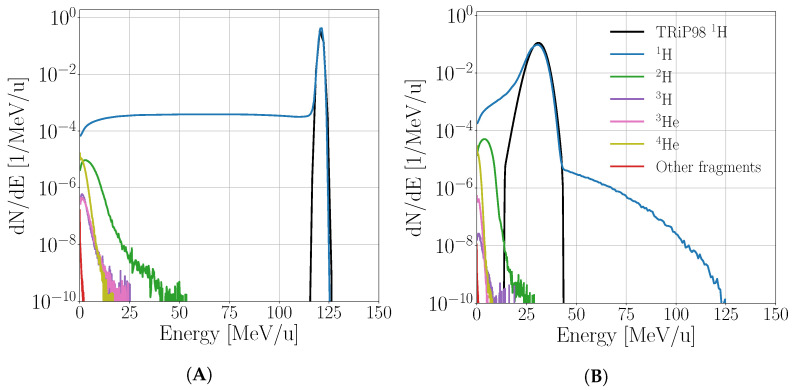
Fluence spectra at 50 mm (**A**) and 158 mm (**B**) depth in semi-logarithmic scale. The black curve represents the currently used spectrum in TRiP98.

- **Physics models**
  Our simulations were based on the default physics model implemented in TOPAS v3.3, namely [35,40]:
  – standard Geant4 Electromagnetic module version opt4
  – high precision QGSP_BIC_HP model
  – ion binary cascade model
  – decay physics model
  – stopping physics model
  – high precision neutron transport model, with G4NDL4.5 data
  Electromagnetic tables were limited to 100 eV–500 MeV range, suitable for particle therapy applications. Range production cuts were set to default values of 50 um for protons, 10 um for alpha particles and 1 um for ions as described in [41].
- **Beam source**
  The particle source was simulated as a pencil beam of mono-energetic protons. In this simplistic case, the beam has no angular emittance and no spatial dispersion, therefore all protons are emitted in the same direction. Initial kinetic energy of primaries ranged from 60 to 230 MeV/u with 5 MeV/u steps.
- **Simulation Geometry**
  The simulation geometry consists into a simple cylinder of radius 210 mm and length *L*, filled with liquid water (density 1 g/cm${}^{3}$). The ionization potential of liquid water was adjusted to 77 eV to be equal to the value used to create the TRiP98 database. The water cylinder was placed in a volume filled with vacuum. The beam source was located on the symmetry axis of a water cylinder, 10 mm before the front wall.
  In order to reduce calculation time, the length *L* of the cylinder was adjusted according to the kinetic energy *E* of primary particles. Specifically, *L* was modified according to the following formula:
  (1) $$L\left(E\right)=a\;\;\cdot \;{\left(\frac{E}{E_{0}}\right)}^{p}+L_{0}$$
  where:
  (2) $$a\;=0.0022\;\mathrm{cm}\;p=1.77\;E_{0}=\;1\mathrm{MeV}\;L_{0}=\;3\;\mathrm{cm}$$
  We are assuming here that *L* is sufficient to simulate proton interactions with matter up to proton range in water (calculated with Bragg-Kleeman rule [42], *a*, *p* and $E_{0}$ in above equations), with the addition of a distal margin of 30 mm (factor $L_{0}$). The distal margin was chosen as the dose in mono-energetic beams drops sharply after reaching maximum. In order to reduce simulation time, dose scoring was disabled in the very distal part, as this region is out of the scope of our study.
- **Scored quantities** For scoring particle fluence *F*, the default *Fluence Volume scorer* was used [35,43]. The standard step-length estimator was used to calculate fluence in a given volume. In a single history (single projectile track), a fluence in volume *V* is calculated by taking into account step lengths $l_{i}$ of particles inside a given volume.
  (3) $$F=\frac{\sum_{i}l_{i}}{V}$$
  Cross-section for the production of secondary particles by protons is small, less than 500 mb for the largest production channel (${}^{4}$He generated by the p + ^16^O reaction). The refore, to achieve convergence, multiple primary protons were simulated (in our work we used 10^9^ primaries for each scenario).
  For each step, the scored fluence was classified into the appropriate bucket among the following categories:
  – *depth z*: the scoring water cylinder was divided into 200 slices of the same thickness along its symmetry axis
  – *particle type A, Z*: only charged ions were considered (protons and other hydrogen isotopes, ^3^He, alpha particles and heavier ions). Particle type was characterized by a pair of numbers: mass number *A* and atomic number *Z*. Isotopes with Z≤17 were scored.
  – *kinetic energy $E_{kin}$*: particle kinetic energy was evaluated at the simulation step and divided into 200 bins. Bins spanned from 0 MeV up to a certain upper limit. To increase the energy resolution, heavier (i.e., oxygen) ions had an upper limit set to only a fraction of the kinetic energy of primary proton.
  – *generation level (gen)*: applied only for protons in order to distinguish primary particles (protons) from secondary, tertiary and higher generations protons.
  Finally, a complete fluence $F(E_{prim},z,T,E_{kin})$ spectrum was generated, being a function of primary proton energy $E_{prim}$, depth *z*, particle type and generation level T={A,Z,gen} and particle energy $E_{kin}$. An example fluence spectra at the depths of 5 cm and 15.8 cm is shown on Figure 1. It should be noted that the energy distribution plotted on Figure 1 are related to the production cross-section, but include also effect of particle transport. This effect is however negligible for recoil nuclei, while for lighter particles with significant range it may be relevant.
  For scoring the dose, the default *Dose scorer* was used, which accumulates the dose deposited by charged ions, electrons and other particles. The dose was scored using the following classification:
  – *depth*: the scoring water cylinder was divided into 200 slices of the same thickness along its symmetry axis
  – *particle type*: charged ions and electrons liberated by them were considered (protons and other hydrogen isotopes, ^3^He, alpha particles and heavier ions). Particle type was characterized by a pair of numbers: mass number *A* and atomic number *Z* and isotopes up to Z≤17 were scored.
  – *generation level*: applied only for protons in order to distinguish primary particles (protons) from secondary, tertiary, and higher generations protons. Dose from each category of protons included the dose delivered by electrons liberated by proton interactions.
  A set of depth dose profiles $D(E_{prim},z,T)$ was generated, being a function of primary proton energy $E_{prim}$, depth *z*, and particle type *T*.
  TOPAS simulation results were saved in binary file format as it provides better numerical precision than default text format.
- **Conversion to TRiP98 file formats**
  Fragment spectra and depth dose profiles are needed in various tasks performed by TRiP98 software, including mainly in biological calculations. Fluence spectra binned by particle energy calculated by TOPAS were converted into normalized energy-fluence spectra (SPC (http://bio.gsi.de/DOCS/TRiP98/PRO/DOCS/trip98fmtspc.html, accessed on 31 July 2021)) which are necessary inputs to TRiP98. Fragment spectra in TRiP98 are handled as the number of particles $N(E_{prim},z,T,E_{kin})$ normalized in such way that N=1 for primary protons at z=0 for all kinetic possible energies $E_{prim}$:
  (4) $$\sum_{E_{kin}}N\left(E_{prim},0,\left\{\left(Z=1,A=1,gen=0\right),E_{kin}\right\}\right)=1$$
  The number of particles *N* can be derived from fluence in the following way to ensure proper normalization (as in the equation above):
  (5) $$N\left(E_{prim},z,T,E_{kin}\right)=\frac{F(E_{prim},z,T,E_{kin})}{\sum_{E_{kin}}F\left(E_{prim},0,\left\{\left(Z=1,A=1,gen=0\right),E_{kin}\right\}\right)}$$
  Files in SPC format contain histograms of particle numbers normalized by bin widths (in kinetic energy, expressed in MeV/u), denoted here as dN/dE (where *E* refers to kinetic energy of a particle at the point of interaction $E_{kin}$ and should not be confused with primary particle energy $E_{prim}$). Depth dose profiles were converted into normalized depth dose profiles distributions (DDD (http://bio.gsi.de/DOCS/TRiP98/PRO/DOCS/trip98fmtddd.html, accessed on 31 July 2021)) also required by TRiP98. Conversion included change of kinetic energy units, as TOPAS operates in MeV while TRiP98 uses MeV/u. Custom converter scripts were created, based on open source pytrip package [44] for Python programming language.

### 2.2. Biological Effect Evaluation

The development (October 2015) version of TRiP98, allowing the simulation of several particle beams including protons, has been used for the study. As a preliminary check, the plan calculated by using the default TRiP98 values that considers only primary protons was compared with the plan obtained by using the look-up spectral data (SPC) generated using TOPAS simulations for primary protons only, to ensure consistency. Then, each plan was calculated for the three cases of study (only primary protons, all protons, all particles) and the outputs are compared in terms of biological and physical dose, RBE and cell survival.

The biological effect is calculated in terms of cell survival probability modeled using a linear-quadratic-linear (LQL) formalism

(6) $$s\left(D\right)=\left\{\begin{array}{cc} e^{-\alpha D-\beta D^{2}} & :D\leq D_{t}\\ e^{-\alpha D_{t}-\beta D_{t}^{2}}e^{S_{m}\left(D-D_{t}\right)} & :D>D_{t} \end{array}\right.$$

where α and β are the intrinsic linear-quadratic parameters, *D* is the dose, $D_{t}$ the threshold dose above which the behaviors turn to be linear and $S_{m}=\alpha 2\beta D_{T}$ is the maximum slope reached from the dose response curve. The se LQ parameters are evaluated using the LEM-IV [45,46] with TRiP98.

#### 2.2.1. Mixed Fields Model

To quantify the radiobiological impact of nuclear fragments, a mixed radiation field approach is used [20,47,48,49]. This is based on LEM-IV and implemented as a standard RBE calculation in TRiP98. A dose averaged $\alpha_{D},\beta_{D}$ are calculated as in Equation (8)

(7) $$\begin{array}{c} \bar{\alpha }={\left(\sum_{l}w_{l}\frac{dE}{dx}\left(l\right)\right)}^{-1}\sum_{l}w_{l}\frac{dE}{dx}\left(l\right)\alpha_{l}\\ \sqrt{\bar{\beta }}={\left(\sum_{l}w_{l}\frac{dE}{dx}\left(l\right)\right)}^{-1}\sum_{l}w_{l}\frac{dE}{dx}\left(l\right)\sqrt{\beta_{l}}\\ w_{l}=\frac{dN}{dE}\left(B_{i},z_{h},T,E\right)N_{spot}\left(B_{i},x_{i},y_{i}\right)\zeta_{ik} \end{array}$$

where the index *l* expands into particle type, *T*, energy, *E*, raster spot *i* (This treatise concerns about active beam delivery, while it can be applied for passive beam delivery with additional ab-initio conditions to account for scattering phenomena [50]) and depth bin *k* · $\frac{dN}{dE}$ denotes the normalized particle spectra, in this work calculated with Equation (5), ΔE the width of a particular spectrum bin and ΔN its content. *N* spot is the number of particles in raster spot *i* (with primary ion energy $B_{i}$ and position $x_{i},y_{i}$, and the $\zeta_{ik}$ are the relative distances of the actual water-equivalent depth of the voxel under consideration to the upper and lower depth-table break-point, respectively [37] and $\frac{dE}{dx}$ is the energy loss calculated by TRiP98 default look up tables for each single particle separately.

#### Mono-Energetic Beams

Firstly, the case of pencil beam is analyzed. In order to study the biological effect of an RBE accounting for the full spectrum, several mono-energetic beam irradiations have been evaluated separately for the tissues and the scoring method described in Section 2.1. A selection of results is reported in Section 3.1.

#### Spread out Bragg Peaks

Two geometrical SOBP cases are considered to reproduce a more clinical-like scenario; a target area of 30 × 30 × 30 mm^3^ was placed at a depth of 160 and 280 mm in water, referred to the box center. A collection of DDD and SPC was created to cover the energy range of 60–230 MeV/u in 5 MeV steps, allowing the system to interpolate for intermediate energies. A selection of results is reported in Section 3.2.

#### Experimental Data Comparison

The survival fraction predicted in this work has been compared with experimental data published in Howard et al. [11], for a mono-energetic proton beam irradiation on T98 cell line, whose α/β ratio was similar enough to our exemplary tissues as described in Section 3.3. Additionally, the RBE evaluated at 50% and 80% of survival (RBE${}_{50}$ and RBE${}_{80}$, respectively) and for different α/β ratios, are compared with experimental data and previous predictions not including the fragments’ prediction, in the case of several SOBP irradiations.

Such experimental data are a collection of 19 publications which have been previously selected in [10] from in vitro experiments for clonogenic cell survival measured along the proton SOBP and grouped based on dose-averaged linear energy transfer (LET${}_{D}$) ranges: 0–1keV/μm and 1–2.5keV/μm [10]. The LET${}_{D}$ values are obtained from the treatment planning system for each position independent on the LET given in the publications considering only primaries. To select the data, the following criteria were applied: the reference radiation is a type of photon radiation and only tumor and normal tissue cell lines were used, the SOBP position and characteristics are clearly described in the publication and the LQ parameters are furnished both for proton beam and reference radiation (more details can be found in [10]). Since the inter-experimental uncertainty was exceeding the intra-experimental uncertainty and the error bars furnished in the original publications were either evaluated in different ways either not provided, the authors of [10] defined the errors from the scatter of the data points, treating each publication with equal weight.

The experimental RBE was therefore calculated applying the Linear Quadratic (LQ) model [51] with the α/β ratio of the photon dose-response curve derived from the cell survival curves after carbon ion irradiation; if the dose level was exceeding the threshold dose $D_{t}$, the LQ curve was assumed to transit into a purely linear shape and thus the linear-quadratic–linear (LQ-L) model [52] was used instead. The RBE values of this work are obtained applying the mixed field approach of TRiP98 in the case of the SOBP at 160 mm, optimized to a flat physical dose in the region of interest as it was used for the experiments. The calculation has been repeated on the same geometry, considering the primary protons’ spectrum only and the ones accounting for the full particle spectrum. The consistency of the comparison between these single data calculations and the averaged values from [10] was validated on the compatibility of the results of primary protons only. In order to quantify the agreement between the different model predictions and experimental data, a Chi-square statistics was adopted for the analysis of the survival curves. At the same time, for RBE data in order to quantify the distance between the linear interpolation of the experimental data (y(α/β)) and the theoretical evaluations (t(α/β)), a euclidean metrics has been used, defined as follows:

(8) $$d^{2}\left(y,t\right)=\int {\left(y-t\right)}^{2}d\left(\alpha /\beta \right)$$

## 3. Results

### 3.1. Mono-Energetic Beams

**Figure 2:**
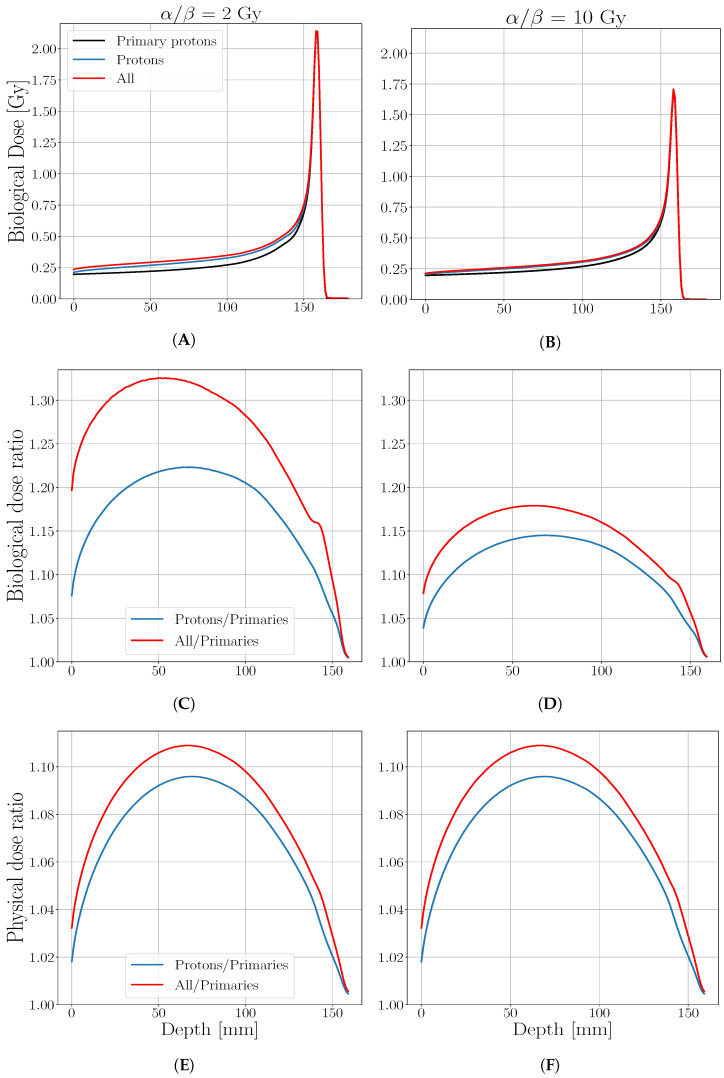
Biological dose as a function of water depth where the black curve is calculated considering the contribution of primary protons only, the blue one of all protons and the red curve all particles (protons and target fragments), (**A**,**B**). The panels (**C**,**D**) report the ratio between the biological dose obtained considering all protons over the biological dose obtained considering only primary protons (blue) along with the ratio between the biological dose obtained considering all produced particles over the biological dose obtained considering only primary protons (red). (**E**,**F**) reports the same ratios but for the physical dose. The cited results are calculated for a tissue characterized by an α/β of 2 Gy, (**A**,**C**,**E**) and α/β of 10 Gy (**B**,**D**,**F**).

A selection of results for mono-energetic beams is reported in Figure 2, where the biological dose is shown as a function of depth for a beam of initial energy 150 MeV/u on a tissue of $\alpha_{x}/\beta_{x}=2$ Gy and 10 Gy, respectively; the biological dose enhancement due to the target fragments contribution is analyzed by evaluating the ratio between the biological dose obtained considering all protons (i.e., primary and secondary protons) and the biological dose obtained considering only primary protons as a function of the depth, compared with the same ratio for the biological dose obtained by considering all particle contributions.

The maximum difference in terms of biological dose is reached in the plateau region (in a range of relative depth of 30–60%) for both curves, where the maximum ratio is 1.33 for all fragments in the case of $\alpha_{x}/\beta_{x}=2$ Gy. Such difference is partially due to the physical dose difference, but it is not the major contribution, as it can be seen from the physical dose ratio reported in Figure 2E,F.

**Figure 3:**
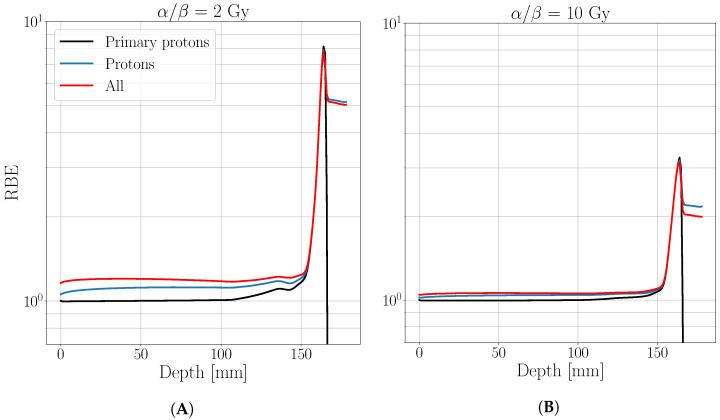
Relative biological effectiveness of a proton beam with 150 MeV/u initial energy obtained by considering the contribution of only primary protons (black), all protons (blue) and all particles (red). The evaluation is reported in semi-log scale for a tissue characterized by an α/β of 2 Gy (**A**) and a tissue characterized by an α/β of 10 Gy (**B**).

The differences found in the biological dose distribution between the three analyzed cases are a direct consequence of the RBE behavior, reported in Figure 3. Primary protons’ RBE remains constant to 1 in the entrance region, as expected, while reaches 1.1 value if secondary protons are considered. Furthermore, a visible enhancement is found in this region if all fragments are considered, especially for the most radio-sensitive tissue (α/β= 2 Gy). As well as the biological dose, RBE value reaches the same maximum values on the BP while a further enhancement in respect to the case where only protons are considered, is found behind the BP position due to secondary protons contribution only.

**Figure 4:**
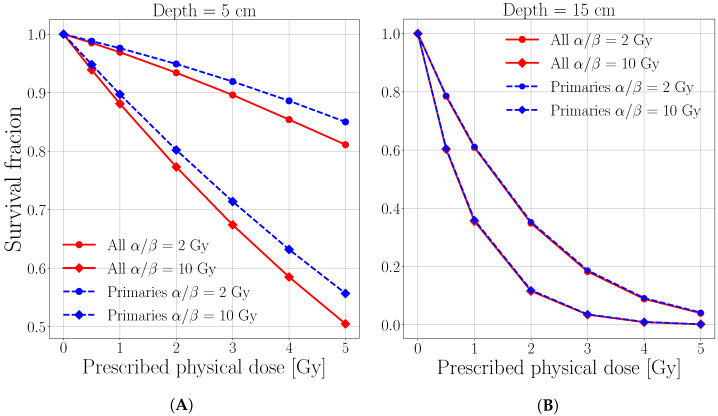
Survival fraction vs. prescribed physical dose at 50 mm (**A**) and 150 mm (**B**) resulting from considering only primary protons (dashed blue curves) and all ions (solid red curves). The circular points represent the values for an exemplary tissue with α/β=2 Gy while the squared points an exemplary tissue with α/β=10 Gy. Please note that the figures are in linear-linear scale to better appreciate the differences between the reported quantities in the high survival range.

The survival fraction calculated by applying the method described in Section 2.2.1 has been investigated at different dose values for several initial energies. A case study for the initial energy of 150 MeV/u in the plateau region (50 mm depth) and on the peak (150 mm depth) is reported in Figure 4. At 50 mm depth, the survival fraction for both tissues is obviously lower when target fragments are considered with respect to the one obtained from primary protons alone. At the same time, in the peak region a saturation effect occurs from the prescribed dose of 5 Gy where the main killing effect is due to slow primary protons. This reflects a negligible impact due to target fragments on the expected survival, independent on the cell radio-sensitivity (Figure 4B).

### 3.2. Spread out Bragg Peaks

**Figure 5:**
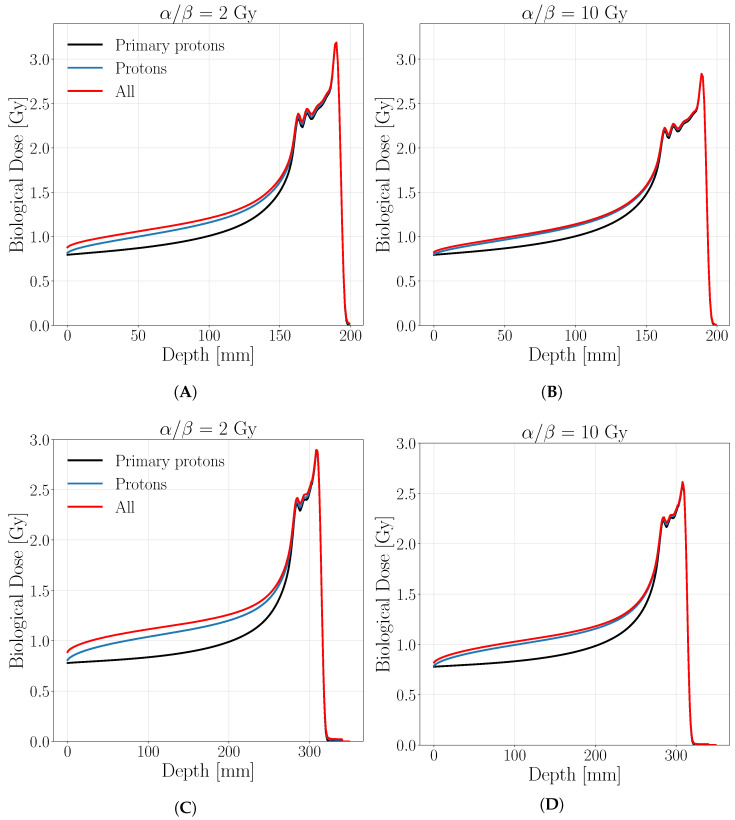
Biological dose for a SOBP calculated with a biological optimization on a target 30 × 30 × 30 mm at a depth of 160 mm (**A**,**B**) and 280 mm (**C**,**D**) for a tissue of α/β=2 Gy (**A**,**C**) and α/β=10 Gy (**B**,**D**). The black line represents the biological dose obtained by considering only primary protons, the blue one is obtained including all protons while in red all protons and target fragment contribution. The legend stands for both panels.

**Figure 6:**
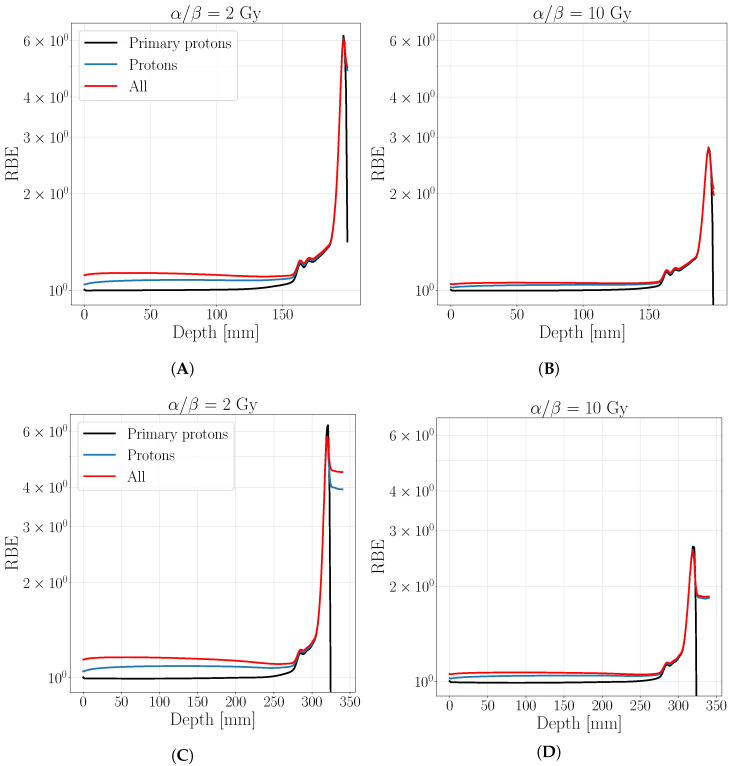
Relative biological effectiveness for a SOBP calculated with a biological optimization on a target 30×30×30 mm at a depth of 160 mm (**A**,**B**) and 280 mm (**C**,**D**) for a tissue of α/β=2 Gy (**A**,**C**) and α/β=10 Gy (**B**,**D**). The black line represents the RBE obtained by considering only primary protons, the blue one is obtained including all protons while in red all protons and target fragment contribution. The legend stands for both panels.

Figure 5 reports the biological dose of the two analyzed SOBP described in Section 2.2.1 obtained with a physical optimization to appreciate the radiobiological differences obtained considering secondaries. The corresponding RBE distribution for the analyzed SOPBs are shown in Figure 6.

There it can be clearly seen that the main contribution, beyond the primaries, is due to secondary protons as well as in the case of mono-energetic beams. The maximum ratio between the biological dose of all particles and the biological dose of only primary protons is found for the most radio-resistant tissue ( α/β=2 Gy) at 1.21 for the shallower SOBP and at 1.33 for the deeper one, both in the entrance region around 50 mm depth. As expected, the secondaries’ contribution to the biological dose in the entrance channel increases with the depth of target and becomes negligible on the peak, as in the previous cases. This behavior is reflected also in the RBE distributions, that remain almost equal to 1 for primary protons in the entrance region, as for the mono-energetic case; if secondaries are accounted a net difference is found in the same area for the tissue of α/β=2 Gy (Figure 6A,C) while it decrease for the tissue of α/β=10 Gy (Figure 6B,D).

### 3.3. Experimental Data Comparison Results

In the following, the RBE and survival fractions evaluated with/without considering the contribution of target fragments, are compared, as a proof of principle, with experimental data and previously published TRiP98-LEMIV predictions based on primary protons’ data only.

**Figure 7:**
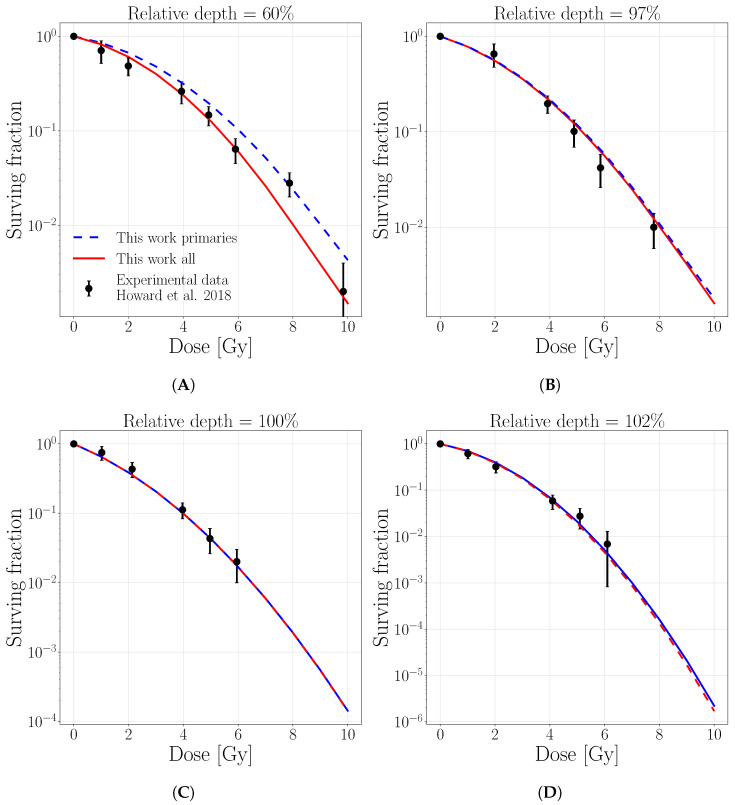
Surviving fraction for T98 cell line as a function of dose for an initial proton beam energy of 71 MeV at the depths of (**A**) 10.1 (60%), (**B**) 16.7 (97%), (**C**) 17.2 (100%), (**D**) 17.5 (102%). Solid lines represent linear interpolations for the surviving fraction evaluated with 1 MeV step, considering the contribution of only primary protons (dashed blue) or all particles (solid red). The legend stands for all panels.

**Figure 8:**
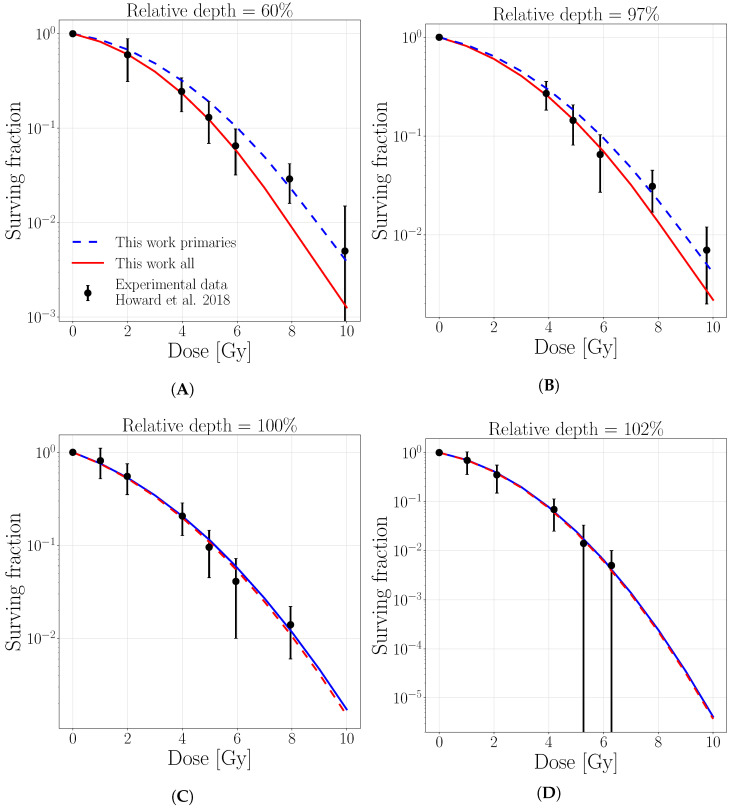
Surviving fraction for T98 cell line as a function of dose for an initial proton beam energy of 160 MeV at the depths of (**A**) 10.1 (60%), (**B**) 16.7 (97%), (**C**) 17.2 (100%), (**D**) 17.5 (102%). Solid lines represent linear interpolations for the surviving fraction evaluated with 1 MeV step, considering the contribution of only primary protons (dashed blue) or all particles (solid red).

The cell survival curves comparisons are reported in Figure 7 and Figure 8 for the T98 cell line (α/β=2.4 Gy, α=0.12 Gy${}^{-1}$, β=0.05 Gy${}^{-2}$). The predictions of this work are tested against experimental data published in Howard et al. [11] for a pristine proton beam irradiation of initial energy of 71 MeV (Figure 7) and 160 MeV (Figure 8). Our calculations consider separately only primary protons and all particles and are evaluated on an exemplary tissue with comparable intrinsic linear-quadratic parameters α/β=2 Gy, (α=0.10, Gy${}^{-1}$β=0.05 Gy${}^{-2}$, D${}_{t}=8\;\mathrm{Gy}$), the closer available data for the comparison. The cell survival fractions as a function of delivered dose, predicted considering the full particle spectra, closely match the experimental data for all the considered depths. Instead, if only primary protons are considered, the agreement is lost in the plateau region, while it is maintained at peak positions. This is also supported by the Chi-square values between experimental data points and predictions with/without taking into account the target fragments, which are reported in Table A1 for 71 and 160 initial beam energies, respectively. The Table A1 indicates that for both initial energies, lower Chi-square values are always attributed to the full-spectra predictions. At the same time, the impact of target fragments gets gradually lower with increasing depth.

**Figure 9:**
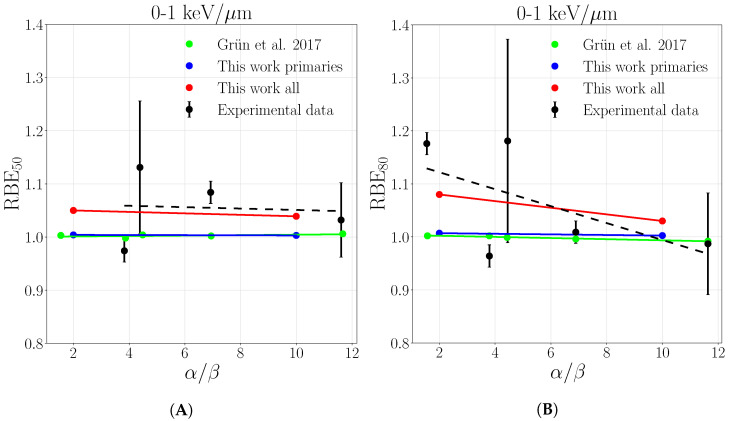
RBE${}_{50}$ (**A**) and RBE${}_{80}$ (**B**) as a function of α/β ratio evaluated in LET range of 0–1 keV/μm. Solid lines report linear regressions for the purpose to guide the reading. Experimental data from Grün et al. [10], the legend stands for both panels.

**Figure 10:**
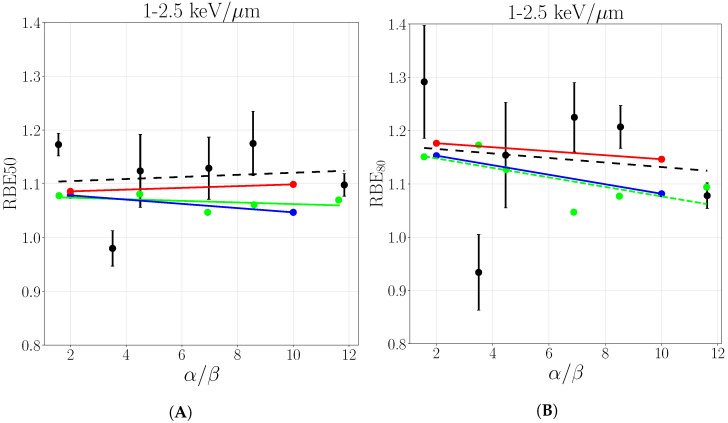
RBE${}_{50}$ (**A**) and RBE${}_{80}$ (**B**) as a function of α/β ratio evaluated in LET range of 1–2.5 keV/μm. Solid lines report linear regressions for the purpose to guide the reading. Experimental data from Grün et al. [10], the legend stands for both panels.

**Figure 11:**
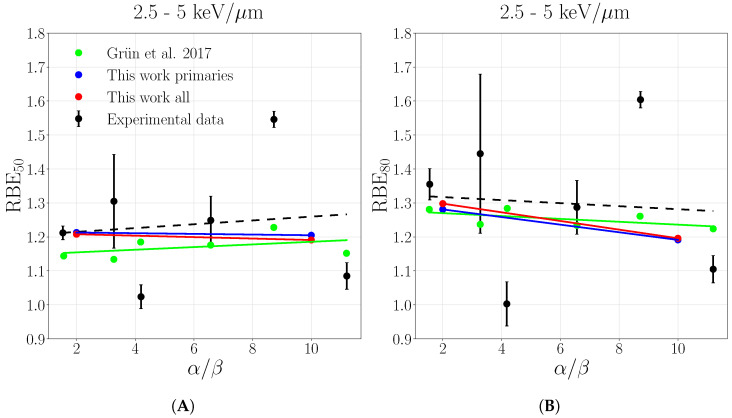
RBE${}_{50}$ (**A**) and RBE${}_{80}$ (**B**) as a function of α/β ratio evaluated in LET range of 2.5–5 keV/μm. Solid lines report linear regressions for the purpose to guide the reading. Experimental data from Grün et al. [10], the legend stands for both panels.

The same behavior is found when RBE${}_{50}$ and RBE${}_{80}$ are evaluated for a spread out Bragg peak and compared with experimental data acquired in three LET${}_{D}$ ranges reflecting the plateau and peak regions, collected in Grün et al. [10]. Figure 9, Figure 10 and Figure 11 show the RBE${}_{50}$ and RBE${}_{80}$, respectively, as a function of α/β ratio where the solid lines are the result of linear regression of the point in correspondent colors, while experimental data are black crosses selected as described in Section 2.2. The consideration of secondaries leads to a better description of experimental values in the LET${}_{D}$ range of 0–1keV/μm (Figure 9) while deviations from the previous primary-based calculations get smaller in the LET range 1–2.5keV/μm (Figure 10), while completely disappear in the highest range (Figure 11). This is supported by the data collected in Table A2. The prediction considering all particles are always closer to experimental data with respect to the ones of only primaries and Grün et al., primary-based calculations, that are similar to each other. An exception is made in the case of RBE${}_{80}$ in the LET range of 1–2.5keV/μm, where all three cases are almost equal, as can be seen in Figure 9. This behavior confirms the trend previously observed, where the difference between only primaries and all particles tends to vanish in the peak region due to the principal role of slow protons. Such comparison offers a more robust proof since it allows to capture the dominant trends of many different data, beyond the experimental fluctuations. The re it is also clearly visible in the lower LET range the amount of the correction with respect to the only primary which is close to 1 as expected and predicted also by Grün et al., offering also a proof of consistency.

## 4. Discussion

The present work aimed at estimating the radiobiological impact of target fragments for proton beams in water for both mono-energetic and SOBP irradiation. While TOPAS was used to describe the physics of the irradiation, the RBE was calculated in TRiP98 by using the implemented mixed field model and LEM-IV, applied to three different sets of spectra: only primary protons, all protons and all particles. This approach allows considering explicitly the secondary particle contribution and directly comparing their impact on RBE, on the contrary to other models based on LET${}_{D}$ [53,54].

**Figure A1:**
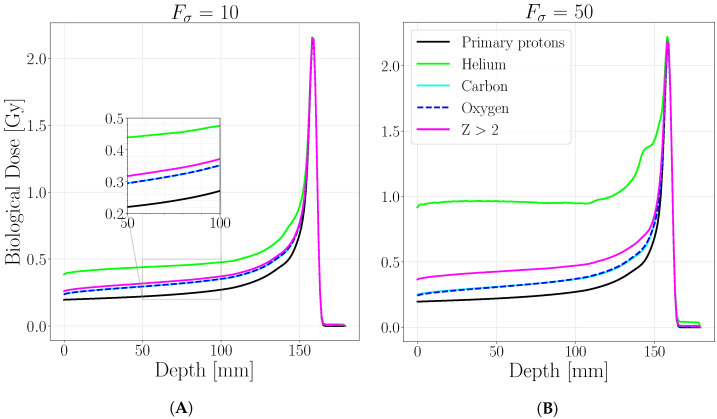
Biological dose distribution in depth of a proton beam with initial energy of 150 MeV/u obtained by considering fluence spectra with a scaled cross-section, $F_{\sigma }=10$ (**A**) and $F_{\sigma }=50$ (**B**), for Helium, Carbon, Oxygen, Z>2. The primary protons’ biological dose is reported without scaling factor to give a direct comparison of the enhancement. The legend stands for both panels.

Our results indicate a non negligible increase in RBE, and therefore in biological dose, associated to target fragmentation. This is principally due to secondary protons and Helium (${}^{4}$He mainly), and is mainly evident in the entrance channel. In fact, the fragments’ RBE contribution reaches its maximum at about 40% relative depth and gradually decreases in the peak region, where the slow primary protons are largely responsible for cell inactivation. As it was already proposed in previous studies [27,33] accounting for secondary protons lead to an enhancement of primary proton RBE which otherwise would be close to 1 as visible in Figure 9, Figure 10 and Figure 11; but the consideration of heavier fragments, particularly ${}^{4}$He is not negligible as shown in Figure A1. This effect is even larger in the entrance region of the considered SOBP where the maximum contribution of fragments is summed. This is a consequence of the secondary particles contributions of the single beams summing up, finally resulting in the regions of maximum fragmentation coming closer to the entrance. The observation that secondary protons and helium fragments are the main contributors to physical dose is in line with what was reported by Grassberger and Paganetti in their MC study [28], which is further complemented here by an explicit RBE evaluation.

While on the one hand this is, to our knowledge, the first analysis aiming at an explicit quantification of RBE due to fragmentation for therapeutic proton beams, on the other hand the results that we provided are affected by the basic uncertainty in the reaction cross-section on which MC codes rely. Initiatives like the FOOT experiment promise providing new and accurate cross-section data in the upcoming years. Based on such data and on the established analysis framework, the impact of target fragments could be easily re-assessed. Meanwhile, some indications can be obtained by the performed sensitivity analysis (reported in Appendix B).

Concerning the comparison to experimental data, the analysis reported in terms of clonogenic cell survival shows that a better agreement with the experimental data by Howard et al. [11] is obtained when the fragments’ contribution is taken into account. This is in fact mainly true for the entrance channel, while the model predictions with/without fragments overlap approaching the peak. This is a consequence of what was already discussed concerning RBE behavior as a function of depth. Remarkably, this is also in line with the analysis by Cucinotta et al. [27] where, based on the Katz model, they were able to describe cell survival after 160 MeV proton beam irradiation only when fragments’ contribution was taken into account. The recent work by Grün et al. [10] was taken as a reference for directly evaluating the impact of target fragments on RBE. The results summarized in Figure 9, Figure 10 and Figure 11 indicate that, compared to the model predictions of the original publication, a slightly improved agreement with the experimental data arises when the full fragments’ spectrum is considered. This is mainly evident in the LET range of 0–1 keV/μm, which correspond to the entrance region. A question could arise whether the LEM-IV model could be applied at such low energies, in particular for the heavier fragments. LEM-IV was indeed verified for a broad energy and LET range [16,55] including mixed field irradiations [56], considering also that every SOBP is a mixed field including stopping and more energetic particles. While it is true that uncertainties on LET and range arise at very low energies, the verification of LEM-IV with different experiments at high LET justify its application also in this case. Moreover, the presented comparison with experimental data, shown in Figure 9, Figure 10 and Figure 11 can be considered as an indirect validation of this approach.

Among the limits of the present approach, there is the fact that particle spectra are still treated similarly as it is done for projectile fragments, i.e., in a “macroscopic” way, not explicitly accounting for the microscopic behavior of low energy fragments.

As a future development, a dedicated work will be focused on the investigation of radio-biological damages at “cell-level”, comparing the predictions based on the dose scored with the standard Monte Carlo technique and averaged on macroscopic volumes with the one obtained by scoring the dose on cell level without averaging, which for such short range fragments (comparable with cell nuclear sizes) lead to different results.

## 5. Conclusions

Based on MC simulations, we quantified the impact of target fragmentation in a simplified proton therapy irradiation scenario for different tissue types, beam configurations, and delivered doses. We show that deviations from an approach including only primary and secondary protons in a clinical plan could become important, mainly for low α/β ratios and for the distal region. We found that the inclusion of full particles spectra provide in general a better agreement with measured RBEs from different pools of experimental data.

## Figures and Tables

**Figure A2:**
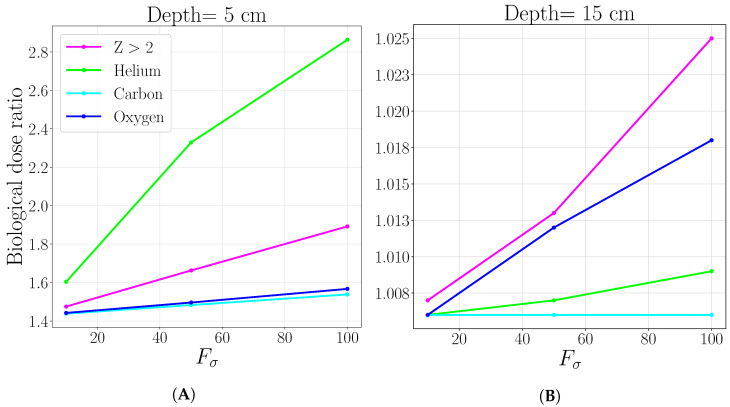
Ratio between the biological dose obtained by considering a modified cross-section for (separately) Helium, Carbon, Oxygen, Z>2 and the biological dose of primary protons, at 50 mm depth (**A**), 150 mm (**B**) as a function of cross-section scaling factor $F_{\sigma }=10,50$ and $F_{\sigma }=100$ as a limit calculation to give an idea of the asymptotic behaviour. The legend stands for both panels.

## Data Availability

Spectra files and depth dose distributions are stored in open access Zenodo.

## Appendix A

A Chi square test has been performed to furnish a relative accuracy level between this work predictions and experimental data of Howard et al. [11] and Grün et al. [10]. The statistical meaning of this test has to be intended as a relative comparison to appreciate which model better describes the experimental data, without expressing the absolute estimation of each models’ accuracy.

The model predictions have been calculated in the experimental data positions and compared with them, considering the errors. Table A1 reports the Chi square values for the comparison between this work prediction, considering all particles or only primary protons, and experimental points of Howard et al. [11] reported respectively in Figure 7 and Figure 8. The test result reflects what can be seen visually from the figures, the prediction considering all the produced particles better represents the experimental data for both the considered energies in the plateau region (relative depth of 60%) and nearly before the BP (relative depth of 97%), while it gives equal accuracy in the remaining regions.

A similar behavior is reflected by a similar statistical analysis carried out between the outcomes of this work and the experimental data published in Grün et al. [10] in the first two LET ranges, since they are the most relevant for the scope of the present work. In this case an equivalent euclidean metrics of Equation (A1), reported here again for a matter of usefulness

(A1) $$d^{2}\left(y,t\right)=\int {\left(y-t\right)}^{2}d\left(\alpha /\beta \right)$$

has been used to quantify the distance between the linear interpolation of the experimental data (y(α/β)) and the theoretical evaluations (t(α/β)); the results are presented in Table A2.

The prediction considering all particles have always lower values with respect to the ones of only primaries and primary-based previous calculations, that are similar to each other. An exception is made in the case of RBE${}_{80}$ in the LET range of 1–2.5keV/μm, where all the three cases are almost equal, as can be seen in Figure 9, Figure 10 and Figure 11.

**Table A1:** Reduced Chi square test values comparing experimental survival data from Howard et al. [11] and predicted values of this work considering all particles and only protons in the case of an initial energy beam of 71 MeV and 160 MeV. The plots reporting the models and the experimental data are respectively Figure 7 and Figure 8.

Relative Depth	Particles	$\chi^{2}$ E = 71 MeV	$\chi^{2}$ E = 160 MeV
60%	All	0.969	0.456
	Primaries	2.656	0.692
97%	All	0.980	0.431
	Primaries	1.311	0.484
100%	All	1.101	0.102
	Primaries	1.095	0.124
102%	All	0.388	0.972
	Primaries	0.414	1.066

**Table A2:** Euclidean metrics outcomes of Equation (A1), comparing experimental survival data from Grün et al. [10] and predicted values of this work considering all particles and only protons in the case of RBE${}_{50}$ and RBE${}_{80}$ (Figure 9, Figure 10 and Figure 11).

LET Range [keV/μm]	Tested Distribution	Euc. Metrics RBE${}_{50}$	Euc. Metrics RBE${}_{80}$
	All	16.473	48.774
0–1	Primaries	17.958	69.477
	Grün et al., primaries	17.805	72.361
	All	30.673	21.981
1–2.5	Primaries	41.419	21.334
	Grün et al., primaries	38.1388	21.2924

## Appendix B. Cross-Section Sensitivity Analysis

The radiobiological impact of the cross-section uncertainties has been studied by evaluating the biological dose based on using a modified cross-section (σ*) scaling for a constant factor the TOPAS produced spectra, σ of the most relevant ions (Helium, Oxygen and Carbon) separately and for all ions with Z>2, in the mono-energetic case:

(A2) $$\sigma^{*}=F_{\sigma }\;\cdot \;\sigma ,\;\;\;\;F_{\sigma }=10,50,100.$$

Figure A1 shows the results of the sensitivity analysis study for the case of an initial energy of 150 MeV/u, as well as a summary of the biological dose values as a function of $F_{\sigma }$ is summarized in Figure A2 for a depth of 50 and 150 mm and for a tissue of α/β=2 Gy. The major impact is found for Helium, reaching a maximum relative difference of 2.1 for $F_{\sigma }=10$ and 5.04 in the case of $F_{\sigma }=50$ in the plateau region. Such multiplication factors should be seen as an indication for the potential impact of an increased CS on the resulting biological dose. In the peak region, instead, the dose of slow protons is predominant and the effect of target fragments is minimized.

The uncertainty of target fragments production cross-section affects mostly the entrance region, differently to nuclear models uncertainties [22] and its impact decreases gradually for smaller and shallower target volumes. This analysis indicates that a stronger impact could arise from an underestimation of the Helium production by at least a factor 2. A previous work [57] investigates in a different situation (distal part of a carbon ion beam) the clinical impact of carbon fragments at very low energy, <500 /; keV/u, by using the TRiP98 transport code computed spectra. The y found a negligible effect of such particles even when the intrinsic RBE is enhanced at those energies by a factor 1000. This result is due to a very low specific weight of those particles in the considered spectra, and for the obvious consideration of the overkill effect. In our case, instead, besides the different irradiation scenario, we are considering not only that extreme energy range but a full distribution arising from the target fragments of a proton beam (Figure 1) obtained by MC calculations. Finally, we perform our sensitivity analysis by scaling the fluence spectra directly and not the RBE.
